# Virus-encoded microRNA contributes to the molecular profile of EBV-positive Burkitt lymphomas

**DOI:** 10.18632/oncotarget.4399

**Published:** 2015-07-31

**Authors:** Pier Paolo Piccaluga, Mohsen Navari, Giulia De Falco, Maria Raffaella Ambrosio, Stefano Lazzi, Fabio Fuligni, Cristiana Bellan, Maura Rossi, Maria Rosaria Sapienza, Maria Antonella Laginestra, Maryam Etebari, Emily A. Rogena, Lynnette Tumwine, Claudio Tripodo, Davide Gibellini, Jessica Consiglio, Carlo M. Croce, Stefano A. Pileri, Lorenzo Leoncini

**Affiliations:** ^1^ Hematopathology Section, Department of Experimental, Diagnostic, and Experimental Medicine, S. Orsola-Malpighi Hospital, Bologna University School of Medicine, Bologna, Italy; ^2^ Department of Medical Biotechnology, University of Siena, Siena, Italy; ^3^ School of Biological and Chemical Sciences, Queen Mary University of London, London, UK; ^4^ Department of Pathology, University of Nairobi, Nairobi, Kenya; ^5^ Department of Pathology, Makerere University, Kampala, Uganda; ^6^ Tumour Immunology Unit, Department of Health Science, Human Pathology Section, Palermo University School of Medicine, Palermo, Italy; ^7^ Department of Pathology and Diagnostic, University of Verona, Verona, Italy; ^8^ Department of Molecular Virology, Immunology, and Medical Genetics, Comprehensive Cancer Center, The Ohio State University, Columbus, OH, USA; ^9^ Diagnostic Hematopathology Unit, European Institute of Oncology, Milan, Italy

**Keywords:** Burkitt lymphoma, miRNA, BART6, EBV, pathogenesis

## Abstract

Burkitt lymphoma (BL) is an aggressive neoplasm characterized by consistent morphology and phenotype, typical clinical behavior and distinctive molecular profile. The latter is mostly driven by the MYC over-expression associated with the characteristic translocation (8;14) (q24; q32) or with variant lesions. Additional genetic events can contribute to Burkitt Lymphoma pathobiology and retain clinical significance. A pathogenetic role for Epstein-Barr virus infection in Burkitt lymphomagenesis has been suggested; however, the exact function of the virus is largely unknown.

In this study, we investigated the molecular profiles (genes and microRNAs) of Epstein-Barr virus-positive and -negative BL, to identify specific patterns relying on the differential expression and role of Epstein-Barr virus-encoded microRNAs.

First, we found significant differences in the expression of viral microRNAs and in selected target genes. Among others, we identified *LIN28B, CGNL1, GCET2, MRAS, PLCD4, SEL1L*, SXX1, and the tyrosine kinases encoding *STK10/STK33*, all provided with potential pathogenetic significance. GCET2, also validated by immunohistochemistry, appeared to be a useful marker for distinguishing EBV-positive and EBV-negative cases. Further, we provided solid evidences that the EBV-encoded microRNAs (e.g. BART6) significantly mold the transcriptional landscape of Burkitt Lymphoma clones.

In conclusion, our data indicated significant differences in the transcriptional profiles of EBV-positive and EBV-negative BL and highlight the role of virus encoded miRNA.

## INTRODUCTION

Burkitt Lymphoma (BL) is an aggressive neoplasm characterized by consistent morphology and phenotype, typical clinical behavior and distinctive molecular profile. The latter is basically determined by *MYC* over-expression, as a consequence of the characteristic translocation (8;14) (q24; q32) or of variant genetic aberrancies. Besides *MYC*, additional genetic lesions can be found, with possible impact on significance [1–4]. Interestingly, despite BL gene expression profile (GEP) is relatively homogeneous and distinct from those of other lymphomas, significant differences have been recorded among the three classical variants, including endemic BL (eBL), sporadic BL (sBL) and immunodeficiency-associated BL (ID-BL) [5]. This finding was also confirmed by microRNA (miRNA) profiling which showed that the three BL variants represent the same biological entity but with marginal differences between endemic and sporadic BL [6].

Of note, the three BL subtypes differ concerning the association with pathogens; in fact, eBL is strictly associated with EBV infection as well as with the exposure to chronic malaria and arbovirus [5, 7, 8]. Conversely, EBV is rarely seen in sBL [5, 7, 8]. EBV has been shown to affect the host cell homeostasis at different levels largely depending on the latent infection type (i.e. latency type). There are three major types of EBV latency that depend on different EBV gene expression patterns and are specific for different cell/disease types. EBNA1 and EBER antigens are expressed in all types of EBV latency, while other latent proteins differ among the diverse latency forms [9]. Latency I configures an *escamotage* for effective EBV immune escape - as the only protein associated with this type of latency, EBNA1, inhibits presentation by MHC-I. Differently, latency type II, which is characterized by the expression of LMP1 and 2, occurs in patients with Hodgkin's lymphomas and T/NK lymphomas, while latency-III, which encompasses expression of all the main EBV proteins are expressed, including EBNA1–2-3, and LMP1–2, is associated with immunodeficiency/immunosuppression states like HIV infection or organ transplantation, or with *in vitro* culturing of latency I cells, like some BL cell lines [10]. Recently, the contribution of different EBV-encoded molecules has been explored in BL and post transplant lymphoproliferative disease [11]. The influence of EBV on host cell transcriptional programs is not only related with the synthesis of latency-related proteins, rather it is also due to EBV interference with host cell miRNA biogenesis and to the synthesis of virus-encoded miRNAs [12]. The EBV genome encodes for 45 mature miRNAs from 25 precursors, which are mapped in 2 regions of the genome: BHRF1 (Bam HI fragment H rightward open reading frame I) and BART (Bam HI-A region rightward transcript) [13]. The BART region encodes the cluster 1 and clusters 2 EBV-miRNAs, whereas the BHRF1 region contains only 3 miRNAs [14]. EBV-encoded miRNAs are differentially expressed among the different latency programs, being the latency III restricted to BHRF1 miRNA expression and the latency I and II to BART miRNA expression [15].

Since the role of EBV in BL pathobiology is still quite debated, and little is known about the influence of EBV-encoded miRNAs in primary BLs, we investigated the miRNA expression profiling of EBV-positive and EBV-negative BL, aiming at identifying differential miRNA patterns according to EBV infection status and at determining the contribution of EBV-derived miRNAs to BL molecular profile.

## RESULTS

### Burkitt lymphomas differ for gene expression and cellular pathway regulation according to EBV presence

**Figure 1:**
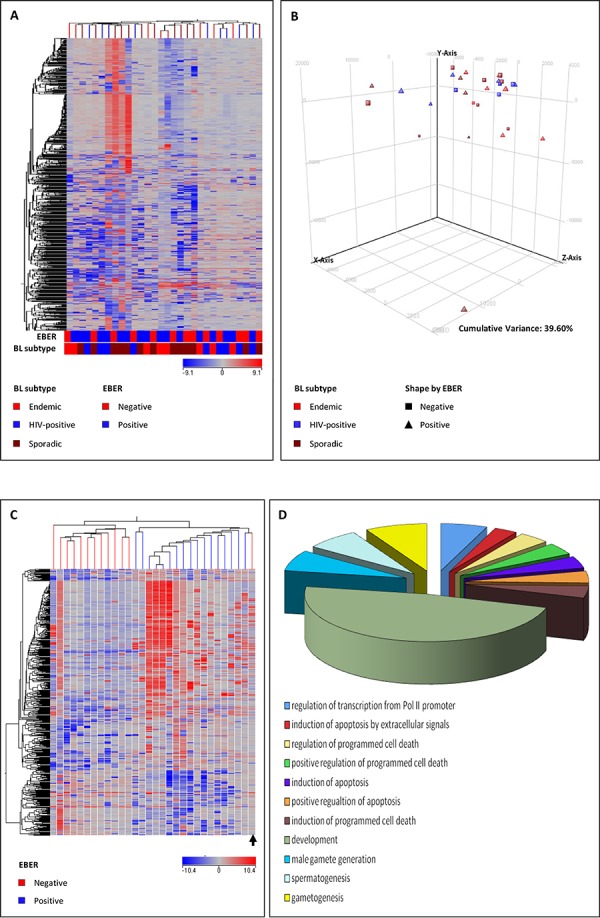
Unsupervised analyses (unsupervised hierarchical clustering, A. principal component analysis, B. failed to clearly discriminate Burkitt lymphoma (BL) subgroups based on the global gene profile Supervised analysis (*T*-test) comparing EBV-positive and EBV-negative cases identified a series of differentially expressed genes **C.** In the matrix (A, C), the dendrogram was generated using a hierarchical clustering algorithm based on the average-linkage method. In the matrix, each column represents a sample and each row represents a gene. The color scale bar shows the relative gene expression changes normalized by the standard deviation (0 is the mean expression level of a given gene). The differentially expressed genes corresponded to significantly enriched biological functions according to Gene Ontology **D.**

First, we aimed to assess whether BL cases differed in gene expression according to the EBV status. Unsupervised approaches confirmed that BL is a rather homogenous disease. In fact, both principal component analysis (PCA) and unsupervised hierarchical clustering (HC) failed to discriminate cases according to either the clinical type (endemic *versus* sporadic *versus* HIV) and the EBV status (positive *versus* negative) (Figure 1A–1B).

However, when supervised analysis was performed (*T*-test, *p* < 0.05, fold change ≥ 2, Benjamini Hockeberg FDR), we could clearly separate EBV-positive and EBV-negative BL based on the expression of 467 genes, differentially regulated in the two subsets. Specifically, 355 genes were up-regulated in EBV-positive cases, while 112 genes were down-regulated (Figure 1C–1D; Supplementary Table 1).

**Figure 2:**
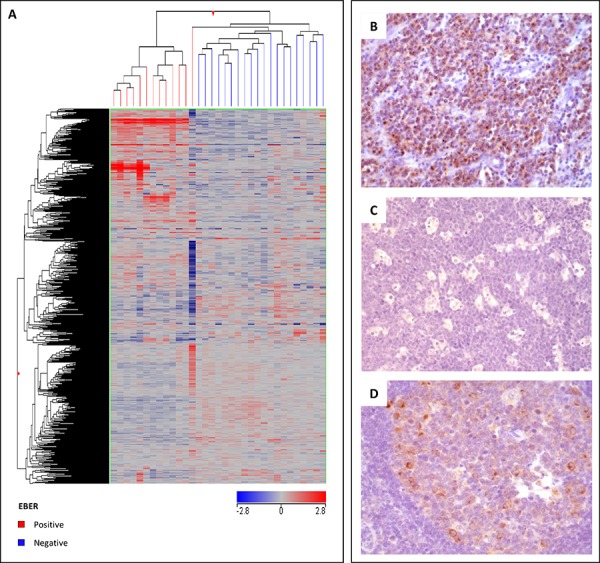
Validation of gene expression profiling **A.** The molecular signature identified as distinctive of EBV-positive *versus* EBV-negative BL was applied to an independent set of cases, allowing a proper distinction of the two groups. In the matrix, each column represents a sample and each row represents a gene. The color scale bar shows the relative gene expression changes normalized by the standard deviation (0 is the mean expression level of a given gene). Immunohistochemical validation of mRNA results confirmed the differential expression of GCET, showing intense positivity in EBV-negative cases **B.** and negativity in EBV-positive cases **C.** which turned out to be strongly meaningful (*p* = 0.0008). As control for immunohistochemistry, a reactive germinal center was used **D.**

To test its validity, this signature was further applied to an independent data set of cases that we previously studied with a different technology (accordingly, the 467 genes corresponded to 858 probe sets in this analysis) [5] and, also in this set of cases, it efficiently separated the EBV-positive and EBV-negative groups (Figure 2A). Similarly, by applying a classification method based on a support vector machine algorithm, 33/34 samples (overall accuracy, 97%) were correctly classified (Supplementary Table 2).

Interestingly, among the most strongly upregulated genes in EBV-positive cases we found *LIN28B*, which is frequently over-expressed in diverse primary tumors, facilitating cellular transformation. Remarkably, *LIN28B* was recently found to serve as key driver gene in HBV-induced carcinogenesis [36]. Further, we found *CGNL1* which encodes for a protein regulating the activity of the small GTPases RHOA and RAC1. Of note, RHOA malfunction due to somatic mutations has been recently described in BL [37, 38]. *GCET2* (alias *GCSAM* or *HGAL*) was also found to be differentially expressed. This was quite interesting. In fact, this gene encodes a protein which may function in signal transduction pathways and whose expression is usually elevated in germinal cell lymphomas and that regulates the RHOA signaling pathway [39].

*SXX1* was also strongly over-expressed in EBV-positive cases; this gene is commonly translocated in synovial sarcomas and renal adenocarcinoma with possible transforming activity [40]. Other genes with potential pathogenetic role include *MRAS, PLCD4, SEL1L*, the apoptosis regulators *CIDEB* and *CRLF3*, the methyltransferase *METTL6* and the serine/threonine kinase encoding *STK10*/*STK33*.

The differential expression of the protein encoded by one of the genes included in the signature, GCET2, was validated at protein level by immunohistochemistry, which turned out to be positive in 5/7 EBV-positive *versus* 2/31 EBV-negative cases (*p* = 0.0008) (Figure 2B).

Further, to make our data more robust, the differential expression of *PLCD4, METTL6, CIDEB* and *CRLF3* was tested and validated in an independent, previously described set of cases, including 13 EBV-positive BL and 20 EBV-negative BL cases [5](Supplementary Figure 1).

We then investigated whether the signature discriminating EBV-positive and EBV-negative BL cases was enriched for genes involved in specific cellular programs and functions and found that EBV-positive cases presented a significant de-regulation of genes related with apoptosis induction, this being in line with the known effects of the virus on B-cells. Most importantly, other programs with potential pathogenetic significance were enriched in EBV-positive BL, including some that are also mediated by known oncogenes such as KRAS, ALK, CCND1, and JNK, and programs related with signal transduction of G protein-coupled receptors (GPCR) (Supplementary Table 3).

We subsequently investigated whether the classification of BL subtypes according to the EBV status was more robust than the clinical classification at the molecular level. To this aim, the molecular signature discriminating sBL and eBL, which we previously identified [5], was applied. According to this signature we could correctly classify all the cases (100% accuracy) as eBL or sBL, independently from the EBV status (Table 1), this indicating that BL arising in different settings, display significant molecular differences that are not recapitulated by the EBV status. Most importantly, this finding demonstrated the actual value of the current clinical classification of BL subtypes. Of note, only 9/467 genes differentially expressed between EBV-positive BL and EBV-negative BL overlapped with those differentially expressed between eBL and sBL (Supplementary Figure 2).

**Table 1:** Classification of BL cases according to the molecular signature discriminating eBL and sBL

Identifier	BL subtype	Predicted BL subtype	Confidence Measure	EBV status
BL_20	Endemic	Endemic	0.9998895	NEG
BL_14	Endemic	Endemic	1	POS
BL_15	Endemic	Endemic	0.999925	POS
BL_17	Endemic	Endemic	0.9999268	POS
BL_16	Endemic	Endemic	0.99990785	POS
BL_18	Endemic	Endemic	0.999925	POS
BL_19	Endemic	Endemic	0.999931	POS
BL_21	Endemic	Endemic	0.9999988	POS
BL_7	Sporadic	Sporadic	0.99992514	NEG
BL_3	Sporadic	Sporadic	0.9998459	NEG
BL_8	Sporadic	Sporadic	0.99990106	NEG
BL_4	Sporadic	Sporadic	0.99993503	NEG
BL_12	Sporadic	Sporadic	0.9998819	NEG
BL_2	Sporadic	Sporadic	0.9998722	NEG
BL_10	Sporadic	Sporadic	0.9999405	NEG
BL_1	Sporadic	Sporadic	0.99991775	NEG
BL_6	Sporadic	Sporadic	0.9998288	POS
BL_5	Sporadic	Sporadic	0.999936	POS
BL_11	Sporadic	Sporadic	0.9998968	POS
BL_9	Sporadic	Sporadic	0.99990225	POS
BL_13	Sporadic	Sporadic	1	POS

### Burkitt lymphomas differ for viral miRNA expression according to EBV presence

Since significant differences were found in the transcriptional profile of BL according to EBV status, we investigated whether miRNA profiles could contribute to this diversity.

**Figure 3:**
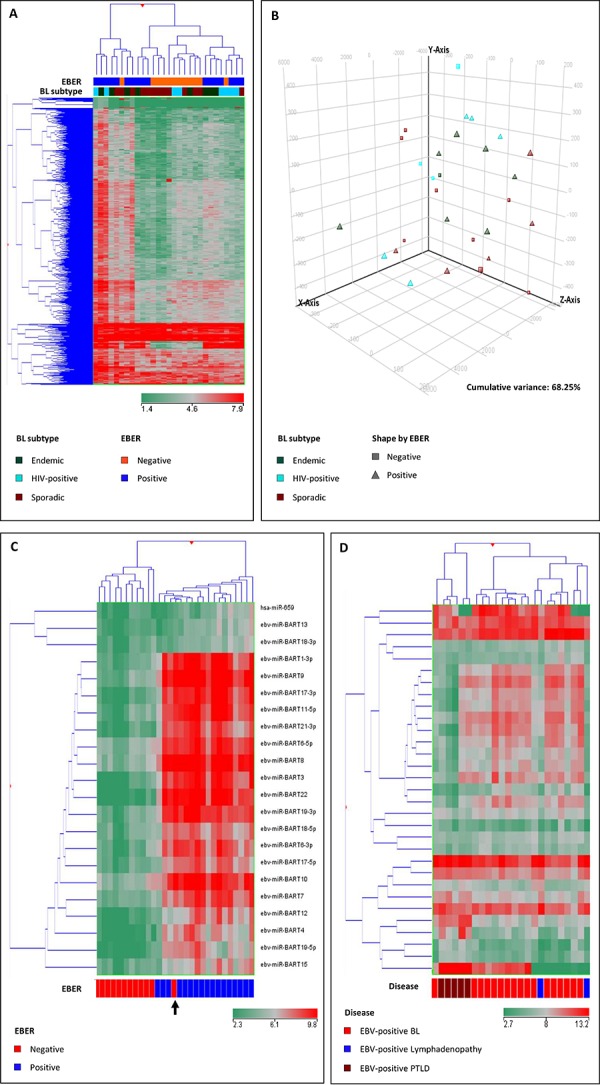
Unsupervised analyses (unsupervised hierarchical clustering, A. principal component analysis, B. failed to clearly discriminate Burkitt lymphoma (BL) subgroups based on the global miRNA profile Supervised analysis (*T*-test) comparing EBV-positive and EBV-negative cases identified a series of differentially expressed miRNAs **C.** The hierarchical clustering of EBV-positive BL, benign lymphadenopathy and post-transplant lymphoproliferative disease (PTLD) indicated differences in regulation of the expression of viral miRNAs in the 3 settings **D.** In the matrix (A, B, C, D), the dendrogram was generated using a hierarchical clustering algorithm based on the average-linkage method. In the matrix, each column represents a sample and each row represents a miRNA. The color scale bar shows the relative gene expression changes normalized by the standard deviation (0 is the mean expression level of a given miRNA).

Similarly to what we observed through analysis of mRNA profiles, by using unsupervised approaches (PCA and HC) to miRNA profiles, we found that all BL subtypes were quite homogeneous, not being clearly discriminated into subgroups (Figure 3A–3B).

When a supervised analysis was adopted to identify specific miRNAs differentially expressed according to the EBV status (positive *versus* negative) of BL cases, we uncovered that one single cellular miRNA, namely hsa-miR-659, differentiated the two groups, while 21 EBV-encoded miRNAs were differentially expressed in the two categories (Figure 3C; Table 2). Of note, one sample, which was initially classified as EBV-negative, clustered within EBV-positive cases (Figure 3C, arrow) as it did at GEP analysis (Figure 1C, arrow). As this case showed expression of viral miRNAs, EBER analysis was repeated and eventually confirmed the EBV presence, though in a small minority of cells. Although anecdotal, this case underscored the reliability of the molecular mRNA and miRNA profiles in discriminating BL cases according to the EBV status.

**Table 2:** MicroRNAs differentially expressed between EBVpos and EBVng BL biopsies, as judged by miRNA profiling

miRNA	*p* Value	Fold change	Regulation in EBV-negative BL
hsa-miR-659	5.216587E-4	3.1564796	down
ebv-miR-BART1–3p	2.2763237E-7	13.949068	down
ebv-miR-BART3	2.2763237E-7	25.136276	down
ebv-miR-BART4	2.2763237E-7	11.487968	down
ebv-miR-BART6–3p	8.566455E-7	9.675229	down
ebv-miR-BART6–5p	3.7144142E-7	8.102603	down
ebv-miR-BART7	1.8088365E-6	7.2138195	down
ebv-miR-BART8	2.2763237E-7	13.425398	down
ebv-miR-BART9	3.3071314E-7	19.268543	down
ebv-miR-BART10	4.381665E-6	7.4831443	down
ebv-miR-BART11–5p	2.2763237E-7	14.604774	down
ebv-miR-BART12	2.2763237E-7	9.735685	down
ebv-miR-BART13	7.181049E-4	2.6553512	down
ebv-miR-BART15	2.0135598E-5	4.21289	down
ebv-miR-BART17–3p	2.2763237E-7	19.245274	down
ebv-miR-BART17–5p	6.5513194E-7	7.0488086	down
ebv-miR-BART18–3p	5.83144E-4	2.412564	down
ebv-miR-BART18–5p	2.3061666E-6	5.701385	down
ebv-miR-BART19–3p	3.7144142E-7	11.179877	down
ebv-miR-BART19–5p	3.7144142E-7	12.813777	down
ebv-miR-BART21–3p	1.1779003E-7	15.055322	down
ebv-miR-BART22	1.1779003E-7	54.28091	down

**Figure 4:**
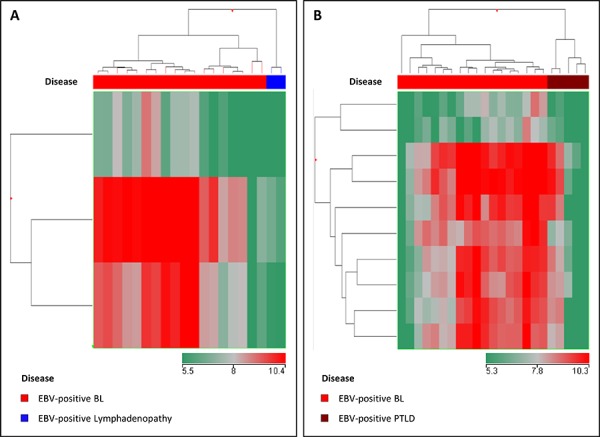
Supervised analysis (Mann-Whitney, *p*-value < 0.05; fold change >2) confirmed differences between EBV-positive BL and EBV-negative positive PTLD A. and EBV-positive lymphadenitis B. in terms of EBV-encoded miRNA. In the matrix (A, B), the dendrogram was generated using a hierarchical clustering algorithm based on the average-linkage method In the matrix, each column represents a sample and each row represents a miRNA. The color scale bar shows the relative gene expression changes normalized by the standard deviation (0 is the mean expression level of a given miRNA).

We then investigated whether the differential viral miRNA signature of EBV-positive BLs was associated either with neoplastic lymphoid transformation or with the type-I viral latency program. To this end, viral miRNA signature was assessed in EBV-positive BL (EBV latency type I), EBV-positive PTLD (latency type III) and benign EBV-positive lymphadenopathy (latency type I). Interestingly, we found that the viral miRNA profile of EBV-positive BL was not related with malignant lymphoid transformation, being different between EBV-positive BL and EBV-positive PTLD. Similarly, it was not related with a specific latency type, being differentially represented in EBV-positive BL and EBV-positive benign lymphadenopathy, both sharing a type-I latency program (Figure 3D). In particular, when EBV-positive BL and EBV-positive PTLD were compared, 10 viral miRNA turned out to be differentially expressed (Figure 4A; Supplementary Table 4), while 3 miRNA differentiated EBV-positive BL and EBV-positive benign lymphadenitis (Figure 4B; Supplementary Table 5). In both instances, viral-miRNAs were up-regulated in BL cases. These results supported the hypothesis that the expression pattern of EBV-related miRNAs in BL is disease-specific and suggested that in BL EBV-encoded miRNA might play a particularly significant role.

### Virus-encoded miRNAs contribute to EBV-positive BL molecular profile

Following the demonstration of a specific EBV-derived miRNA signature characterizing EBV-positive BLs, we investigated whether such EBV-related miRNAs exerted an actual influence over the transcriptional profile of BLs.

**Figure 5:**
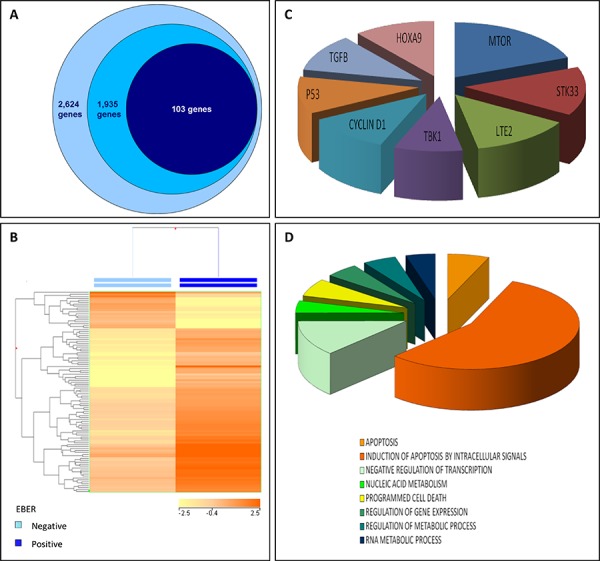
EBV-encoded miRNA significantly impact on BL gene expression profile **A.** Among the 2,624 genes so far demonstrated at biochemical level to be targeted by EBV-encoded miRNAs, 1, 935 are targeted by miRNAs over-expressed in EBV-positive BL. Among these, we found 103 to be differentially expressed between EBV-positive and EBV-negative BL cases. **B.** Based on the expression of such 103 genes, EBV-positive and EBV-negative BL cases were clustered. These 103 genes were involved in oncopathways mastered by known oncogenes **C.** and biological processes related to the regulation of apoptosis, transcription and nucleic acid metabolism **D.**

To investigate the possible roles of such miRNAs, we then explored their ability to affect GEP of the tumors. First, we retrieved the list of all EBV-encoded miRNA target genes biochemically proved so far (http://crdd.osdd.net/servers/virmirna/). From this list, we extracted the genes targeted by viral miRNA over-expressed in EBV-positive BL. One hundred three of them were indeed differentially expressed in EBV-positive *versus* EBV-negative BL. (Figure 5A–5B, Supplementary Table 6). This evidence was also confirmed in an independent series of cases for which GEP had been previously generated by our group (data not shown) (5).

Interestingly, at GSEA the differentially expressed miRNA targets tuned out to be significantly involved in regulation of transcription and gene expression, nucleotide/RNA metabolism, and most often apoptosis. Moreover, they could be related to some pathways often involved in tumorigenesis such as those controlled by TP53, TGFB, CCND1, TBK1, LTE2, MTOR, STK33, and HOXA9 (Figure 5C–5D; Supplementary Table 7).

**Figure 6:**
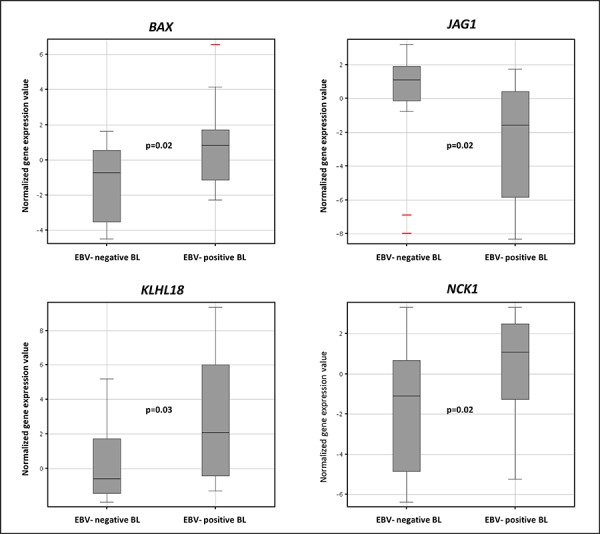
The expression levels of BAX, JAG1, KLH18, and NCK1 in Burkitt lymphoma according to EBV presence Box plot correspond to normalized gene expression values; bars indicate median values as well as 95% confidence intervals. Two tails unequal Student *T*-test was used for comparisons.

Among others, we noted – for the potential pathobiological significance, the differential expression of *BAX*, involved in apoptosis regulation; *JAG1*, the physiological ligand of NOTCH1; *KLHL18*, a ligase that regulates mitotic entry and ubiquitylates Aurora-A; and *NCK1*, one of the signaling and transforming proteins containing Src homology 2 and 3 (SH2 and SH3) domains involved in transducing signals from receptor tyrosine kinases to downstream signal recipients such as RAS (Figure 6).

**Figure 7:**
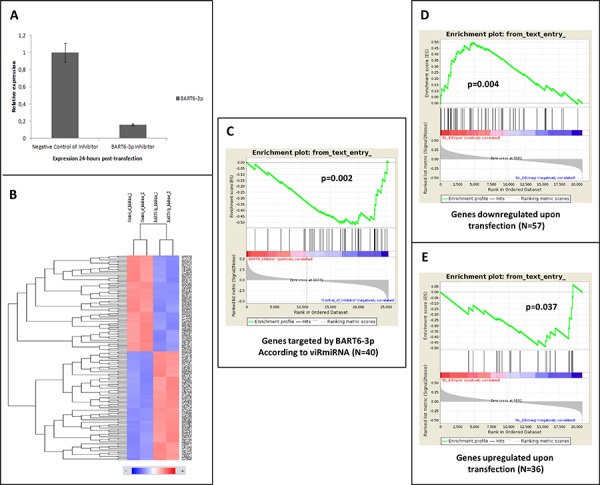
A. Transfection efficiency of AKATA cell lines with BART6 inhibitor was confirmed by quantitative RT-PCR **B.** Gene expression profiles were generated in AKATA cell lines transfected with BART6 inhibitors and negative controls. Supervised analysis of the two groups revealed differential expression of 93 unique genes. **C.** The expression of BART6 targets previously validated at biochemical level was tested upon transfection. Indeed, we found a highly significant enrichment that confirmed the accuracy of the tool. **D–E.** Gene set enrichment analysis documented as the genes differentially expressed in AKATA cell line upon BART6 inhibition were significantly enriched in primary cases supporting the functional role of BART6 *in vivo*.

In addition, an ad hoc functional experiment was performed to investigate the specific effects of the prototypical EBV-derived miRNA BART6–3p. The Akata cell line with the BART6–3p inhibitor, and the effects on such inhibition on the global transcriptome were analyzed. We found that BART6–3p knocking-down (verified by q-PCR, Figure 7A), led to the modified expression of a set of genes, which included 86 induced probes and 76 repressed probes, corresponding to 36 and 57 unique genes, respectively (Figure 7B, Supplementary Table 8).

Noteworthy, GSEA consistently demonstrated the differential expression of the genes previously demonstrated to be targeted by BART6–3p according to viRmiRNA and those identified by us (Supplementary Table 8), between the transfected Akata cells and the primary EBV-positive and EBV-negative subsets (Figure 7C–7E) respectively, further supporting the direct role of EBV-encoded miRNAs in the modulation of BL gene expression profile.

## DISCUSSION

Since its identification as tumor-associated virus in Burkitt lymphoma, EBV has been implicated in the development of a wide range of lymphoproliferative disorders, also including Hodgkin lymphoma and post-transplant lymphoproliferative disease [41, 42]. However, the exact mechanism of action of EBV in human cancer, and especially in BL, is still debated [43–45]. Although the number of the products encoded by EBV in its latent states is very limited, during its evolution the virus has gained the capability of interfering with different cellular physiologic mechanisms which could explain the virus's contribution to hman malignancies. [4, 12, 46–50]. The abovementioned EBV-encoded products include 9 latent proteins, two RNA molecules without known coding properties (i.e. EBERs) and at least 40 miRNA molecules, most of which belong to BART family. Furthermore, these molecules are not expressed equally in different conditions where the virus occurs to be present, and different viral latency types are defined on the basis of the expression pattern of the latent proteins [9, 51–53].

The lack of the expression of the viral oncoproteins like LMP-1 in BL has cast doubt about the role of EBV in BL. In fact, BL shows the latency type I of EBV, where the only viral latent protein expressed EBNA-1 [12, 54]. However, there are several documents, both *in vitro* and *in vivo*, which support such a role. One classical example to be mentioned is the study performed by De-The et al. in 1978 in Uganda: the children who later developed BL showed a higher titer of antibodies against EBV [55]. In addition, results of a study of deep sequencing of primary BL cases showed higher mutation rates in EBV-negative cases, indicating a possible substituting role of EBV for those mutations [56, 57]. Accordingly, Vereide et al. showed that although the infected cells might lose the virus *by chance*, the infected malignant cells seem to depend upon the virus, as they maintain it for apoptosis inhibition [58]. In all, although EBV does not appear to be able to induce BL malignant phenotype by itself as oncogenic activation (typically *MYC* translocation and over-expression) is anyway necessary, EBV is currently considered to facilitate B cell transformation in BL tumorigenesis by inducing a clonal expansion of an apoptosis resistant population [59, 60].

As EBV may contribute to BL pathogenesis with both genes and miRNA, in this study we performed an extensive gene and miRNA expression profile of BL cases carrying or not EBV aiming to evaluate for the first time the influence of virus-encoded miRNA on the global molecular profile of the tumor.

We first proved, for the first time, that BL can be differentiated by gene expression profiling based on the presence of EBV. This is not trivial, as it indicates that the presence of EBV determines significant effects on the cellular programs of the transformed B-cell that are maintained even when the full lymphomatous phenotype is acquired. In this regard, it should be noted that genes differentiating EBV-positive and EBV-negative BL are significantly involved in apoptosis regulation, confirming that cell death impairment might be at least in part mediated by the virus in BL cells. Previous GEP studies actually indicated that BL subtypes have a largely common profile, different from the ones of other lymphomas and that eBL, sBL, and ID-BL can be distinguished based on the gene expression signature [5, 16, 25, 61].

In this regard, intriguingly, when we tried to understand whether the distinction of BL according to the EBV status was more robust than the clinical categorization, this implying a possible new classification system, we found that the clinical setting maintained a significant impact. In fact, by applying a previously identified molecular signature able to discriminate eBL and sBL [5], we could correctly classify all the cases independently from the EBV status. This proved that BL arising in different settings, beside EBV status, have significant molecular differences and confirmed the value of the current classification. Consistently, only few genes were in common between the two signatures.

The evidence of significant differences in EBV-positive and EBV-negative cases at GEP, prompted us to investigate whether viral miRNA could contribute to this phenomenon as miRNA and global gene expression have never been integrated in this setting. We found significant differences in the miRNA profile of tumors carrying or not EBV. However, consistent with a previous report [6], we failed to identify significant differences concerning cellular miRNA, while viral miRNA designated the different patterns. Of interest, only a subset of viral miRNA turned out to be expressed in EBV-positive cases, largely corresponding to the BART family. By contrast, no miRNA from the BHRF regions were expressed. This observation was consistent with the latency I program typical of BL, and appeared to confirm confirming previous reports on EBV-encoded miRNA expression in EBV-associated tumors [62]. However, to further dissect this issue, we evaluated the expression pattern of viral miRNA in cases of non malignant EBV-positive lymphadenitis, characterized by the same latency I program as well as in PTLD, an EBV-positive lymphoid malignancy characterized by a latency III program. We could identify clear differences between BL and PTLD, which also expressed molecules belonging to the BHRF family. However, surprisingly, we found significant differences between BL and non neoplastic samples as well, this indicating for the first time that the EBV-encoded miRNA expression pattern is not univocally dependent on the latency type.

In the last years, an increasing number of publications have stressed the role of EBV-encoded miRNAs in the patho-biology EBV-related tumors, with anti-apoptotic properties as the major indication [11, 63–65]. Very interestingly, it seems that BL cells totally depend upon BART miRNAs in order to exhibit their tumor-related properties [58]. In this regard, we showed that BART6-3p, an EBV-encoded miRNA, may exert growth-inducing properties and affect immune response and impact the global gene expression profile of EBV-positive BL, when compared to EBV-positive PTLD [11, 54]. Here, as a proof of principle, we demonstrated that BART6-3p might affect the gene expression profile of EBV-positive BL at global level, when compared to EBV-negative BL. A similar effect for other BART miRNAs could be assumed, as demonstrated by us in the case of EBV-positive ID-BL [66], however it must be noted that the miRNA-miRNA interactions might be needed to be interpreted in a complex network in which the outcome of the co-expression of several miRNAs be different from simple sum of their effects, due to possible synergism/antagonism among them [67].

In conclusion, our study provided for the first time evidences that EBV-positive and EBV-negative BL have a distinct GEP and that EBV-encoded miRNAs significantly affect BL molecular phenotype by opening new scenarios in the study of BL and other lymphomas and more generally of EBV-related diseases.

## MATERIALS AND METHODS

### Case collection

We collected 30 BL cases from different Italian and African institutions, including 8 endemic, 13 sporadic and 9 immunodeficiency-related BLs, corresponding to 13 EBV-positive and 17 EBV-negative cases. Furthermore, 5 EBV-positive post-transplant lymphoproliferative disease (PTLD) and 2 EBV-positive benign lymphadenopathy cases were included as control (Table 3). The diagnosis was made by at least 3 expert hematopathologists and confirmed as previously described [5, 16].

**Table 3:** Sample description for the tumor and normal biopsies used for miRNA profiling

Samples	Disease	Subtype	EBER	EBV latency
BL_001	BL	Sporadic	NEG	NA
BL_002	BL	Sporadic	NEG	NA
BL_003	BL	Sporadic	NEG	NA
BL_004	BL	Sporadic	NEG	NA
BL_007	BL	Sporadic	NEG	NA
BL_008	BL	Sporadic	NEG	NA
BL_010	BL	Sporadic	NEG	NA
BL_012	BL	Sporadic	NEG	NA
BL_020	BL	Endemic	NEG	NA
BL_023	BL	Immunodeficiency-related	NEG	NA
BL_024	BL	Immunodeficiency-related	NEG	NA
BL_025	BL	Immunodeficiency-related	NEG	NA
BL_030^*^	BL	Immunodeficiency-related	NEG	NA
BL_005	BL	Sporadic	POS	Type I
BL_006	BL	Sporadic	POS	Type I
BL_009	BL	Sporadic	POS	Type I
BL_011	BL	Sporadic	POS	Type I
BL_013	BL	Sporadic	POS	Type I
BL_014	BL	Endemic	POS	Type I
BL_015	BL	Endemic	POS	Type I
BL_016	BL	Endemic	POS	Type I
BL_017	BL	Endemic	POS	Type I
BL_018	BL	Endemic	POS	Type I
BL_019	BL	Endemic	POS	Type I
BL_021	BL	Endemic	POS	Type I
BL_022	BL	Immunodeficiency-related	POS	Type I
BL_026	BL	Immunodeficiency-related	POS	Type I
BL_027	BL	Immunodeficiency-related	POS	Type I
BL_028	BL	Immunodeficiency-related	POS	Type I
BL_029	BL	Immunodeficiency-related	POS	Type I
PTLD_031	PTLD	DLBCL	POS	Type III
PTLD_032	PTLD	DLBCL	POS	Type III
PTLD_033	PTLD	DLBCL	POS	Type III
PTLD_034	PTLD	DLBCL	POS	Type III
PTLD_035	PTLD	DLBCL	POS	Type III
MNC_036	Lymphadenopathy	Lymphadenopathy	POS	Type I
MNC_037	Lymphadenopathy	Lymphadenopathy	POS	Type I

* Not used in miRNA profiling.

NA: not available

### Gene expression profiling of primary Burkitt lymphomas

Gene expression profile analysis was carried on by using the DASL whole genome assay starting from formaldehyde-fixed, paraffin-embedded (FFPE) tissues [17–22]. RecoverAll™ Total Nucleic Acid Isolation Kit (Life Technologies, Monza, Italy) was used to extract total RNA from FFPE tissues. Up to five 10 μm sections were processed per reaction. FFPE samples were deparaffinized using a series of xylene and ethanol washes. Next, they were subjected to a rigorous protease digestion with an incubation time tailored for recovery of total RNA. RNA was purified using a rapid glass-fiber filter methodology that includes an on-filter DNase treatment and were eluted into the low salt buffer provided. RNA was quantified using NanoDrop spectrophotometer.

Further, total RNA was converted to cDNA using biotinylated oligo (dT) and random nonamer primers. The biotinylated cDNA was then annealed to the DASL Assay Pool (DAP) probe groups that contain oligonucleotides specifically designed to interrogate each target sequence in the transcripts. As these probes span about 50 bases, it is possible to profile partially degraded RNA. Following this, correctly annealed, assay-specific, oligos were extended and ligated to generate amplifiable products. These templates were labeled during PCR amplification by including fluorescent primers in the reaction. The resulting PCR products were scanned using the BeadArray Reader or iScan System to determine the presence or absence of specific genes.

Gene expression analysis was carried on as previously reported [5, 23–25]. Unsupervised clustering was generated using a hierarchical algorithm based on the average-linkage method [26, 27]. Only genes displaying a twofold average change in the expression level across the whole panel were chosen to generate the hierarchical clustering. The expression value of each selected gene is normalized to have a zero mean value and unit standard deviation. The distance between two individual samples was calculated by Pearson correlation with the normalized expression values. To perform the supervised gene expression analysis, we used GeneSpring GX 12 (Agilent, MI, Italy) [5]. Differentially expressed genes between different groups were identified using a two-tails Student *t*-test and adjusted Benjamini-Hochberg correction for false discovery rate, applying the following filtering criteria: *p*-value <0.05, and fold change >2. A Support Vector Machine algorithm was used for sample classification as previously reported [5, 28].

EASE software was applied in order to establish whether specific cell functions and biological processes, defined according to gene ontology [29, 30], were significantly represented among the deregulated genes [31, 32].

Gene set enrichment was calculated with the use of Gene Set Enrichment Analysis (GSEA) [33] with *t* test–based *P* values for weighting statistics.

Gene expression analysis was carried on according to MIAME guidelines. Raw gene expression data will be available at http://www.ncbi.nlm.nih.gov/projects/geo/ after publication (GSE63665).

### miRNA profiling of primary Burkitt lymphomas

MicroRNA expression profiling was performed using Nanostring nCounter^®^ miRNA Expression Assay Kits (Human V1 miRNA NanoString Technologies, Seattle, WA, USA)… Raw data coming from nCounter^®^ miRNA Expression Assay were normalized using NanoStringNorm package developed in R 2.15 version. Briefly, probe levels quantified by microarrays were adjusted for miRNAs with specific background correction factor. Technical normalization was performed using geometric mean of positive controls and mean of negative controls for background subtraction. Lastly, the dataset was normalized such that the mean of each gene is zero. The data were further analyzed using GeneSpring GX12. The miRNAs differentially expressed between the two categories were selected on the basis of the following criteria: fold change ≥ 2, corrected *p*-value (Benjamini-Hockeberg FDR) ≤ 0.05 [34].

As a source for experimentally validated EBV-encoded miRNA targets, we used viRmiRNA (http://crdd.osdd.net/servers/virmirna/), a recently established database which in addition covers Experimental viral miRNAs and Experimental antiviral miRNAs of a wide range of human and non-human viruses [35].

### Cell transfection and gene expression analysis

For functional validation experiments, the EBV-positive BL Akata cell line was transfected with BART6–3p inhibitor or a negative control of inhibitor (NCI, Dharmacon-Thermo Scientific, Germany) using Amxa Nucleofector apparatus (Amaxa, Cologne-Germany), program G23 and transfection solution V, according to the manufacturer's instructions. RNA was extracted 24 hours post-transfection, and the transfection efficiency was confirmed by q-PCR by means of Taqman probes (Applied Biosystems, Germany), employing RNU43 as housekeeping miRNA, as described previously [12]. The expression levels were calculated by ΔΔCT method utilizing NCI sample as control. RNA was further processed according to the manufacturer's instructions and hybridized on the HuGene-2.0-st array (Affymetrix, Santa Clara, CA). HuGene-2.0-st CEL files were quantile normalized and log2 transformed using rma method implemented by the Bioconductor R package for Affymetrix as previously reported [5, 23–25]. Supervised analysis (two-tails student *T*-test) was performed to obtain molecular signatures of different cell lines using geWorkbench 2.4.1 software and filtered using the following criteria: *p*-value < 0.01, fold change ≥ 3.

Enrichment in expression of BART6–3p predicted and experimentally found targets was evaluated using GSEA software on a set of 12 EBV-positive endemic, 2 immunodeficiency-related and 21 sporadic BL samples which were extracted from GEO database and normalized as described above [5].

The study was conducted according to the principles of the Helsinki declaration after approval of the Internal review Board.

### Immunohistochemistry

GCET2 immunohistochemical staining was performed as follows: 2–4-μm-thick paraffin-embedded tissues were cut onto Dako slides (DAKO), and subsequently dewaxed, rehydrated and subjected to antigen retrieval by heating in 50 mM Tris (tris(hydroxymethyl)aminomethane) (Trizma base)-2 mM EDTA (ethylenediaminetetraacetic acid) (Sigma Chemical, St Louis, MO) (pH 9) in a microwave pressure cooker (A Menarini Diagnostics, Wokingham, UK) at 900 W for 2 minutes. The slides were cooled and treated with peroxidase-blocking solution (DAKO) for 5 minutes. Sections were then immunostained with Gcet1 mAb by the two-stage peroxidase-based EnVision technique (DAKO), counterstained with hematoxylin and mounted.

Incubations containing unrelated antibodies were used as a control of the technique. The difference of the expression level between the cases was evaluated using two-tailed Fisher's exact test

## SUPPLEMENTARY FIGURES AND TABLES


