# Supplementary material for: Virus-encoded microRNA contributes to the molecular profile of EBV-positive Burkitt lymphomas
Source: Oncotarget. 2015 Jul 31;7(1):224–40. doi: 10.18632/oncotarget.4399 (PMC4807994; doi:10.18632/oncotarget.4399)
Supplement: Supplementary file 1 [file oncotarget-07-0224-s001.pdf]

SUPPLEMENTARY FIGURES, AND TABLES

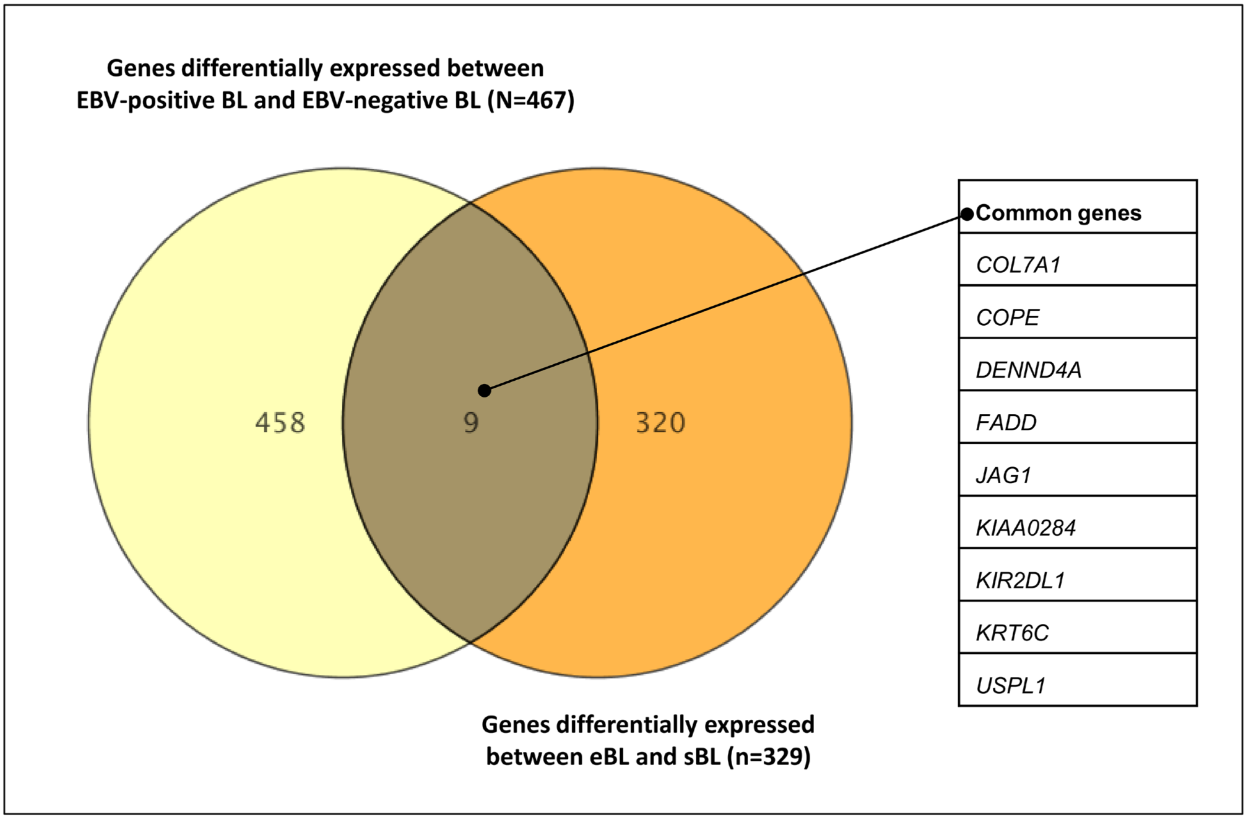

Supplementary Figure S1: Validation of gene expression data in an independent dataset including 13 EBV-positive BL and 20 EBV-negative BL cases. Gene expression values were obtained by Affymetrix HG U133 2.0 plus. Two tails unequal Student *T*-test was used for comparisons.

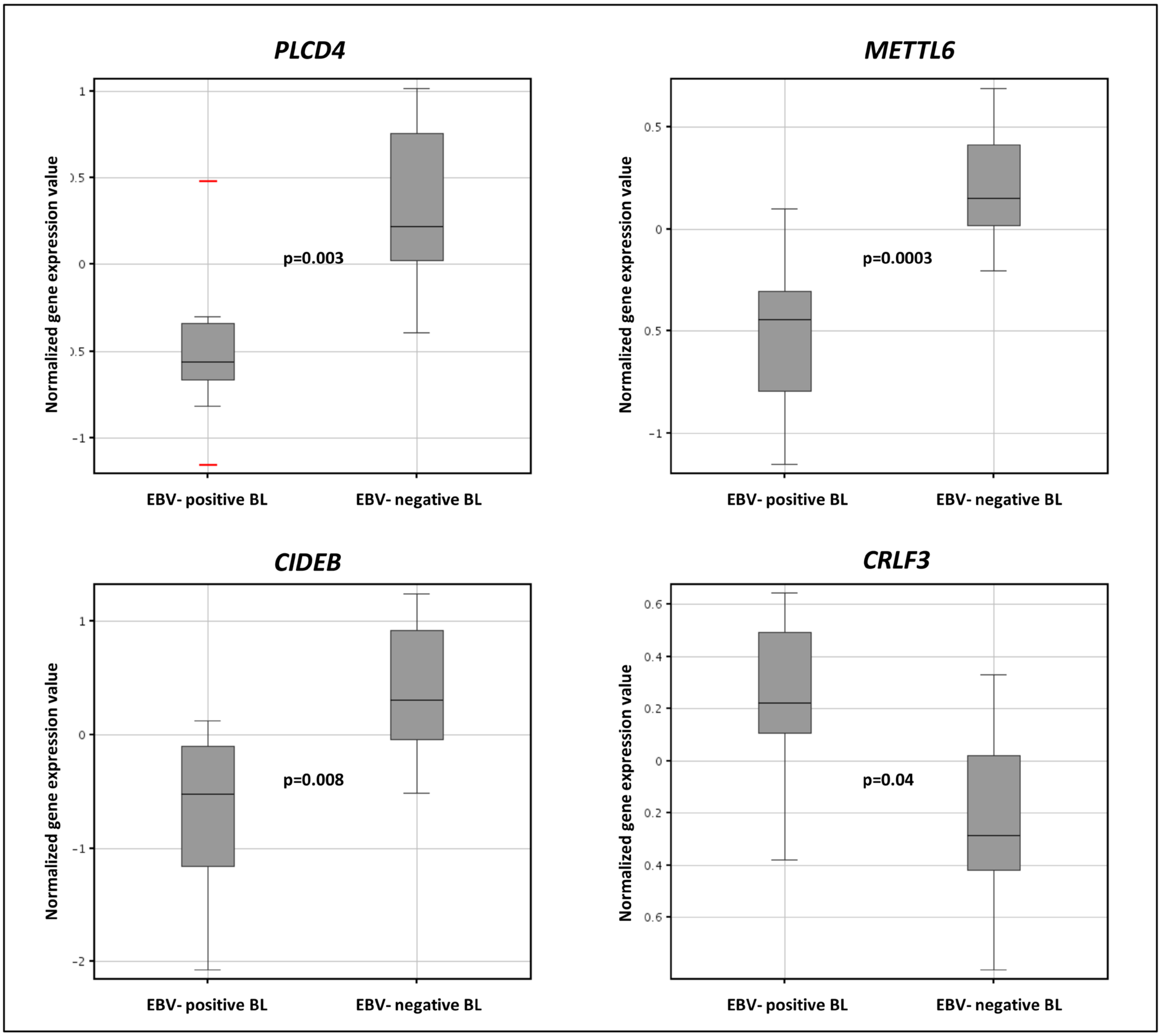

Supplementary Figure S2: Venn diagram showing the overlap between the two molecular signatures differentiating EBV-positive BL vs. EBV-negative BL (*N* = 467) and eBL vs. sBL (*N* = 329).

Supplementary Table S1. Genes differentially expressed in EBV+ vs. EBV- BL cases (*T*-test, $p < 0.05$; Folde change > 2)

TargetID	Fold change	Regulation in EBV+
ALDH18A1	8.272479	DOWN
ANKRD57	4.757337	DOWN
ARRB1	3.9845333	DOWN
ATP5F1	4.1921687	DOWN
BAP1	3.1505635	DOWN
C11ORF60	8.28758	DOWN
C12ORF44	2.8071501	DOWN
C16ORF68	12.292065	DOWN
C17ORF79	2.6983845	DOWN
C1ORF54	3.8017142	DOWN
C20ORF46	4.905921	DOWN
C4ORF27	13.500782	DOWN
C6ORF125	7.8422284	DOWN
CAPN12	5.441035	DOWN
CCDC16	2.5386107	DOWN
CDK8	13.569048	DOWN
CHST13	5.8238807	DOWN
CIDEB	12.584771	DOWN
CNPY4	2.2888982	DOWN
COMTD1	6.793182	DOWN
COPE	2.8348198	DOWN
CTXN1	5.3330235	DOWN
CUX1	4.6025376	DOWN
CYBA	7.147015	DOWN
DAP	8.716893	DOWN
DAP3	5.875716	DOWN
DBP	6.808462	DOWN
DBR1	2.9578853	DOWN
DOPEY1	16.890066	DOWN
ELF4	4.503868	DOWN
FADD	6.16555	DOWN
FAM113A	9.720205	DOWN
FBXO36	8.698004	DOWN
FHOD1	3.7963984	DOWN
GATM	18.50725	DOWN

(*Continued*)

TargetID	Fold change	Regulation in EBV+
GGPS1	2.4459949	DOWN
GIYD1	3.945078	DOWN
GLRX	4.4480667	DOWN
GNG5	5.067239	DOWN
GPR135	6.3469377	DOWN
GPRIN1	7.1790485	DOWN
GPX4	2.7978706	DOWN
GSTK1	4.289849	DOWN
HES6	8.8397	DOWN
HEXIM2	11.749028	DOWN
HMOX1	9.468657	DOWN
HSBP1	5.0673947	DOWN
HSDL1	10.838059	DOWN
IMMT	2.5251095	DOWN
IRX5	6.3418336	DOWN
ITGAE	4.3267384	DOWN
JAG1	7.14392	DOWN
JOSD2	7.754276	DOWN
JUNB	11.037125	DOWN
KIAA0195	5.9482493	DOWN
KIAA0261	10.146835	DOWN
LMO7	9.278552	DOWN
LPAR5	4.436608	DOWN
LZIC	11.104232	DOWN
MAD1L1	8.277236	DOWN
MARK3	3.7337978	DOWN
MCAM	5.1313004	DOWN
METTL3	5.556122	DOWN
MRPL21	3.178807	DOWN
MRPL53	16.618849	DOWN
MTIF2	3.0359027	DOWN
MTMR15	7.064877	DOWN
MYH3	14.333212	DOWN
NCF4	2.777556	DOWN
NDUFA7	10.401297	DOWN
NIPSNAP3B	4.920272	DOWN
NUP160	9.537094	DOWN

(*Continued*)

TargetID	Fold change	Regulation in EBV+
OCIAD2	3.1305737	DOWN
OSTF1	7.785394	DOWN
PDLIM7	3.5382667	DOWN
PDZD2	12.824701	DOWN
PDZK3	16.051739	DOWN
PIH1D1	5.408546	DOWN
PITPNB	2.8292985	DOWN
PNKP	3.651734	DOWN
PRKCSH	2.547611	DOWN
PSMA3	2.9827435	DOWN
PTCH1	9.69102	DOWN
RCN3	11.9623	DOWN
RDH5	3.6980836	DOWN
RINL	6.1802974	DOWN
RIOK2	8.061623	DOWN
RND2	4.207678	DOWN
RNF220	9.683698	DOWN
SAMM50	6.6760097	DOWN
SELM	4.686855	DOWN
SERPINE1	4.5133843	DOWN
SH2B1	7.593262	DOWN
SHD	11.533251	DOWN
SLC19A3	9.633501	DOWN
SLC35D2	11.983629	DOWN
SMARCC1	7.415126	DOWN
STARD5	6.7760696	DOWN
TMED10	5.3114476	DOWN
TMEM134	2.8511553	DOWN
TRIB1	4.1135073	DOWN
TTC1	3.952104	DOWN
TWISTNB	7.0502	DOWN
TYROBP	15.176877	DOWN
UBXN6	5.623031	DOWN
UNC45A	3.3507986	DOWN
USO1	4.553762	DOWN
USP5	7.9540215	DOWN
WDR81	13.588858	DOWN

(*Continued*)

TargetID	Fold change	Regulation in EBV+
XYLT2	5.5482483	DOWN
ZFAND2B	4.038632	DOWN
ZFYVE26	4.2463813	DOWN
ABCF2	3.3022532	UP
ABHD4	10.155282	UP
ABTB2	5.19592	UP
ACSL3	2.3765633	UP
ADAM11	9.452753	UP
ADAMTS2	5.496341	UP
AKAP11	3.4643428	UP
AKT1	2.2303436	UP
ALG11	4.318087	UP
ANKMY1	3.9011917	UP
ANKRD12	5.086308	UP
AP2S1	3.8783805	UP
AP4M1	10.40215	UP
ARL15	3.9619644	UP
ARL5B	4.868017	UP
ARMC4	11.461546	UP
ARV1	4.5958295	UP
ASMT	4.976675	UP
ASTL	6.568938	UP
B3GAT3	14.016187	UP
B4GALT5	10.572061	UP
BAI3	7.9501247	UP
BCAS3	7.5588794	UP
BEX1	7.967559	UP
BIVM	7.4948916	UP
BPTF	4.5908523	UP
BRCA1	3.0345447	UP
BRMS1	3.9091043	UP
BSN	7.6498375	UP
BTBD1	2.218938	UP
BTG1	4.14162	UP
BTK	2.50178	UP
C10ORF79	6.4710827	UP
C11ORF76	5.484194	UP

(*Continued*)

TargetID	Fold change	Regulation in EBV+
C11ORF82	6.7545958	UP
C15ORF5	3.1798685	UP
C17ORF65	14.282438	UP
C17ORF77	6.921021	UP
C18ORF19	7.012164	UP
C19ORF15	5.3405995	UP
C19ORF48	3.4147694	UP
C2CD2L	6.3411975	UP
C2ORF65	7.754112	UP
C3ORF63	10.7603655	UP
C4ORF36	7.862698	UP
C6ORF199	6.3262286	UP
C8ORF15	4.1638856	UP
C9ORF3	6.88235	UP
C9ORF66	8.562933	UP
CAPZA3	10.616991	UP
CASP2	3.016799	UP
CASP5	8.52568	UP
CBFB	3.236214	UP
CCDC104	2.5386226	UP
CCDC146	12.73467	UP
CCDC64	6.7159133	UP
CCDC85A	12.848363	UP
CCDC89	6.7066603	UP
CCKAR	4.9335303	UP
CCL11	9.104318	UP
CCS	2.3090925	UP
CDC42SE2	3.0621438	UP
CDKN1A	3.8472886	UP
CDKN2AIPNL	2.8431215	UP
CECR1	4.516518	UP
CGNL1	18.731842	UP
CH25H	8.97794	UP
CIZ1	10.391165	UP
CKM	5.779003	UP
COL7A1	10.301249	UP
COX6A2	5.899649	UP

(*Continued*)

TargetID	Fold change	Regulation in EBV+
CRLF3	3.2298124	UP
CSTA	6.2634993	UP
CUGBP1	3.666287	UP
CXXC4	9.26937	UP
DAND5	5.8151145	UP
DAPL1	11.345971	UP
DBNDD1	4.6581984	UP
DENND4A	2.4383268	UP
DHFR	5.216262	UP
DJ222E13.2	6.121086	UP
DKFZP564N2472	11.058964	UP
DLG2	6.0596547	UP
DLX4	5.731343	UP
DNAHL1	7.085851	UP
DNAJB2	3.9600842	UP
DPM2	6.30971	UP
DYRK2	3.7778282	UP
ECHDC3	16.236204	UP
EDN2	10.148479	UP
EID2B	2.6650562	UP
EPM2AIP1	5.4095006	UP
EXOC5	11.402861	UP
FAM105A	3.258073	UP
FAM169B	6.0863066	UP
FAM54A	3.203376	UP
FANCF	17.171618	UP
FAP	7.0805087	UP
FBF1	3.716196	UP
FBXO6	8.568514	UP
FBXW7	2.431355	UP
FCHO2	8.743793	UP
FCN3	3.014177	UP
FCRL1	9.102244	UP
FCRLB	16.85491	UP
FGD1	12.716766	UP
FGFR2	5.214902	UP
FHL5	10.0654125	UP

(*Continued*)

TargetID	Fold change	Regulation in EBV+
FKTN	3.1157274	UP
FLJ35767	9.513234	UP
FLJ35848	6.048168	UP
FLJ35894	8.661615	UP
FLJ40453	4.5874133	UP
FLJ42953	6.1622906	UP
FLJ46257	5.825679	UP
FLJ46838	7.635382	UP
FNDC8	23.953186	UP
FOXD3	8.569016	UP
FRZB	3.5591276	UP
FXN	3.2622313	UP
FXYD5	2.071927	UP
FZD3	8.65198	UP
GABRG3	5.126418	UP
GABRR1	7.0519533	UP
GBP6	4.9147367	UP
GCET2	3.0690074	UP
GIF	4.6857495	UP
GJB2	6.8092318	UP
GPR120	8.607794	UP
GREB1	3.9330535	UP
HIP1	8.16208	UP
HISPPD2A	3.4400265	UP
HIST1H1T	11.230007	UP
HSCB	2.7309966	UP
HSF2	4.567313	UP
HSPA12B	8.35278	UP
HSPB9	4.7719283	UP
HYLS1	6.8131814	UP
IL1A	9.460081	UP
IL2RB	4.109601	UP
IMPA1	3.1556294	UP
INHBC	10.166702	UP
IQGAP1	5.1078644	UP
IRX1	2.9024196	UP
ITGB3	7.285979	UP

(*Continued*)

TargetID	Fold change	Regulation in EBV+
KCNF1	8.30115	UP
KIAA0101	2.2337723	UP
KIAA0232	5.3072824	UP
KIAA0284	8.130848	UP
KIAA0415	8.788113	UP
KIAA1009	5.9804964	UP
KIAA1432	9.543548	UP
KIAA1543	7.5322566	UP
KIR2DL1	6.6557546	UP
KLK1	3.6086092	UP
KLRG1	3.8868334	UP
KRT1	23.670687	UP
KRT14	18.934446	UP
KRT6C	9.8162365	UP
KRTAP10–9	6.6906514	UP
KRTAP13–4	6.9817095	UP
KRTAP26–1	5.1615777	UP
KRTAP4–1	5.0074816	UP
LAD1	13.795049	UP
LCE1D	5.186705	UP
LCN12	4.3468757	UP
LILRB1	4.753883	UP
LIMD2	3.5856042	UP
LIN28B	49.501858	UP
LMAN2	2.072059	UP
LOC151121	6.0847425	UP
LOC283174	13.093498	UP
LOC389118	4.4606147	UP
LOC644613	6.436031	UP
LOC723972	6.796686	UP
LOH3CR2A	7.8951597	UP
LPA	5.079673	UP
LRAP	5.6914525	UP
LRRC14	2.5199628	UP
LY6G6D	7.62794	UP
MAGEB18	5.875588	UP
MAST4	7.707886	UP

(*Continued*)

TargetID	Fold change	Regulation in EBV+
MDM4	4.9741435	UP
ME2	2.320154	UP
METTL6	4.4296536	UP
MGAT5	8.162192	UP
MGC16291	10.671934	UP
MGC33407	13.686721	UP
MOBKL1A	2.6076138	UP
MPP3	5.2590303	UP
MPP4	5.791646	UP
MPZL1	3.8082228	UP
MRAS	16.658098	UP
MRE11A	2.5959651	UP
MRPL3	3.9280987	UP
MS4A15	7.655296	UP
MSC	7.20591	UP
MTHFD2	2.3979664	UP
NDUFA4L2	9.13538	UP
NLRC5	35.40025	UP
NMU	16.61036	UP
NOG	13.671498	UP
NOTCH2NL	2.5240633	UP
NRG4	10.560629	UP
NRGN	6.636515	UP
NRN1L	5.701939	UP
NUDT9P1	10.528118	UP
OR10H1	7.8625774	UP
OR10J3	6.7213416	UP
OR1N2	6.498315	UP
OR2S2	5.8038173	UP
OR2T35	5.929727	UP
OR4N2	7.1657376	UP
OR4Q3	4.030717	UP
OR52H1	6.336197	UP
OR5H2	7.3992286	UP
OR5H6	5.610058	UP
OR5L2	7.4221644	UP
OR6C74	5.516263	UP

(*Continued*)

TargetID	Fold change	Regulation in EBV+
OR6Y1	6.755625	UP
OTOP3	5.973361	UP
PAQR3	10.292417	UP
PAQR6	3.8678403	UP
PARS2	6.2633934	UP
PCDHA4	5.1748385	UP
PCDHB12	5.5363045	UP
PDIA2	5.160482	UP
PGR	5.766926	UP
PHC3	4.3200583	UP
PHF20L1	2.2821264	UP
PKHD1	4.5833573	UP
PKP1	4.9123435	UP
PLAG1	4.612025	UP
PLCD4	16.007902	UP
PLEKHG1	3.3825002	UP
POGZ	3.470346	UP
POLL	10.348884	UP
PSCDBP	3.2682562	UP
PTP4A3	4.0867376	UP
PTPRA	2.4859471	UP
RAB5A	10.350054	UP
RAC1	2.388142	UP
RACGAP1	2.1135058	UP
RAD51AP1	2.6928227	UP
RAG1	8.1245165	UP
RASGEF1B	10.695118	UP
RBAK	11.6936865	UP
RBM38	3.4565268	UP
RBMY1B	8.926077	UP
RBMY1E	6.4444976	UP
RBMY2FP	4.2365174	UP
RCHY1	2.9764302	UP
RCOR3	3.8835475	UP
RNASE8	4.762706	UP
RNF166	6.101951	UP
RNF180	6.3359623	UP

(*Continued*)

TargetID	Fold change	Regulation in EBV+
RPA4	7.727459	UP
SBSN	5.1126227	UP
SC5DL	3.6928701	UP
SCGB1A1	15.332985	UP
SCGB1D4	6.749087	UP
SEL1L	15.255204	UP
SERPINB13	12.427637	UP
SFI1	3.7482495	UP
SGCG	6.6195984	UP
SGCZ	5.3696847	UP
SIAH2	4.226047	UP
SIGLEC1	12.860411	UP
SIRT4	6.784535	UP
SLAMF6	3.22041	UP
SLC15A4	6.0615964	UP
SLC1A3	8.722222	UP
SLC22A11	9.294568	UP
SLC30A6	3.347841	UP
SLC35E4	9.261727	UP
SLC5A8	3.04959	UP
SLC6A18	6.8505616	UP
SLC6A9	3.176562	UP
SLC7A14	7.141905	UP
SLC9A4	3.2009828	UP
SMAD1	10.658414	UP
SMARCC2	2.7324996	UP
SMR3B	10.649572	UP
SNTG2	5.2343884	UP
SOLH	19.032837	UP
SPANXC	5.550966	UP
SPATA13	7.6829424	UP
SPATA4	4.2678676	UP
SPEN	7.469406	UP
SPRR2B	14.395799	UP
SPRY3	9.175199	UP
SRD5A1	6.3402987	UP
SSX1	18.633776	UP

(*Continued*)

TargetID	Fold change	Regulation in EBV+
ST3GAL4	9.608029	UP
STH	6.6100607	UP
STK10	4.4307985	UP
STK33	3.849843	UP
TAAR2	4.712044	UP
TAS2R16	7.4161763	UP
TCBA1	9.42274	UP
TCERG1	2.4149494	UP
TCN1	5.1663337	UP
TERF1	2.163468	UP
TEX11	3.1643672	UP
TGFB2	8.353513	UP
TKTL1	5.717334	UP
TMED2	9.113536	UP
TMEM154	2.7697082	UP
TMEM20	6.633979	UP
TMEM39B	4.6726947	UP
TMEM86B	8.433345	UP
TMPRSS11D	7.0737605	UP
TMSB4Y	14.212225	UP
TNP2	5.465167	UP
TNS3	12.532465	UP
TPCN1	4.2674093	UP
TPTE	6.483994	UP
TRAM1	8.247869	UP
TRHDE	5.2583632	UP
TRIP11	3.3953404	UP
TRPA1	5.832572	UP
TSPAN14	9.823884	UP
TTC21B	3.4910824	UP
TTPAL	3.6855366	UP
TTTY14	5.736401	UP
TTTY2	5.620001	UP
TTTY8	11.351613	UP
UAP1L1	3.5256782	UP
UBL3	8.784547	UP
UBTD1	7.9808	UP

(*Continued*)

TargetID	Fold change	Regulation in EBV+
UCN3	9.483514	UP
UCP2	6.9032526	UP
UGT3A2	6.984359	UP
UNC13C	5.395858	UP
USMG5	7.5987515	UP
USP6	7.0937643	UP
USPL1	6.447994	UP
VGLL1	3.8508434	UP
VWC2	4.4691696	UP
WASL	6.6322126	UP
XKR3	7.954668	UP
ZCCHC11	4.243349	UP
ZMYM6	5.48934	UP
ZMYND19	5.360929	UP
ZNF135	8.8639765	UP
ZNF385C	10.995965	UP
ZNF414	10.197204	UP
ZNF431	7.658877	UP
ZNF471	5.023195	UP
ZNF493	3.1094515	UP
ZNF530	5.9484353	UP
ZNF543	3.9892805	UP
ZNF69	2.480991	UP
ZNF780B	2.9471548	UP
ZNF826	9.291265	UP

Supplementary Table S2. Classification of an independent set of cases according to the molecular signature discriminating EBV+ and EBV–BL cases

GEO data set	Identifier	Actual EBV status	Predicted EBV status	Confidence Measure
Series GSE26673	BL_001_SI.CEL	EBV+	EBV+	1
Series GSE26673	BL_002_SI.CEL	EBV+	EBV+	1
Series GSE26673	BL_003_SI.CEL	EBV+	EBV+	1
Series GSE26673	BL_004_SI.CEL	EBV+	EBV+	1
Series GSE26673	BL_005_SI.CEL	EBV+	EBV+	1
Series GSE26673	BL_006_SI.CEL	EBV+	EBV+	1
Series GSE26673	BL_007_SI.CEL	EBV+	EBV+	1
Series GSE26673	BL_008_SI.CEL	EBV+	EBV+	1
Series GSE26673	BL_009_SI.CEL	EBV+	EBV+	1
Series GSE26673	BL_010_SI.CEL	EBV+	EBV+	1
Series GSE26673	20091102_38_Pileri_Plus2.0_(HG-U133_Plus_2).CEL	EBV+	EBV+	1
Series GSE26673	20091102_43_Pileri_Plus2.0_(HG-U133_Plus_2).CEL	EBV+	EBV+	1
Series GSE26673	20091102_57_Pileri_Plus2.0_(HG-U133_Plus_2).CEL	EBV+	EBV–	1
Series GSE4732	BL_U133+_2405_68967.cel	EBV–	EBV–	1
Series GSE4732	BL_U133+_2406_67254.cel	EBV–	EBV–	1
Series GSE4732	BL_U133+_2418_68975.cel	EBV–	EBV–	1
Series GSE4732	BL_U133+_2420_67256.cel	EBV–	EBV–	1
Series GSE4732	BL_U133+_2430_67257.cel	EBV–	EBV–	1
Series GSE4732	BL_U133+_2432_67424.cel	EBV–	EBV–	1
Series GSE4732	BL_U133+_2433_67258.cel	EBV–	EBV–	1
Series GSE4732	BL_U133+_2438_68971.cel	EBV–	EBV–	1
Series GSE4732	BL_U133+_2443_68974.cel	EBV–	EBV–	1
Series GSE4732	BL_U133+_2448_67259.cel	EBV–	EBV–	1
Series GSE4732	BL_U133+_2461_69770.cel	EBV–	EBV–	1
Series GSE4732	BL_U133+_2464_69968.cel	EBV–	EBV–	1
Series GSE4732	BL_U133+_2466_69771.cel	EBV–	EBV–	1
Series GSE4732	BL_U133+_2472_67260.cel	EBV–	EBV–	1
Series GSE4732	BL_U133+_2521_68968.cel	EBV–	EBV–	1
Series GSE4732	BL_U133+_2522_67264.cel	EBV–	EBV–	1
Series GSE4732	BL_U133+_2524_67261.cel	EBV–	EBV–	1
Series GSE4732	BL_U133+_2536_67423.cel	EBV–	EBV–	1
Series GSE4732	BL_U133+_2537_67262.cel	EBV–	EBV–	1
Series GSE4732	BL_U133+_2538_67263.cel	EBV–	EBV–	1

Supplementary Table S3. GSEA analysis carried on genes differentially expressed in EBV+ vs. EBV- BL cases revealed significant enrichment in several programs and pathways

GSEA Category	Gene Set Name	Description	#Genes in Overlap	FDR *q*-value
Canonical Pathways	REACTOME_SIGNALING_BY_GPCR	Genes involved in Signaling by GPCR	21	4.53E-03
	REACTOME_GPCR_DOWNSTREAM_SIGNALING	Genes involved in GPCR downstream signaling	19	4.55E-03
	BIOCARTA_PTDINS_PATHWAY	Phosphoinositides and their downstream targets.	4	7.52E-03
	PID_ERA_GENOMIC_PATHWAY	Validated nuclear estrogen receptor alpha network	5	2.49E-02
	PID_ERBB1_DOWNSTREAM_PATHWAY	ErbB1 downstream signaling	6	2.49E-02
	KEGG_REGULATION_OF_ACTIN_CYTOSKELETON	Regulation of actin cytoskeleton	8	3.57E-02
	SIG_CHEMOTAXIS	Genes related to chemotaxis	4	4.48E-02
	PID_DELTANP63PATHWAY	Validated transcriptional targets of deltaNp63 isoforms	4	4.48E-02
	BIOCARTA_ATM_PATHWAY	ATM Signaling Pathway	3	4.48E-02
	REACTOME_ENOS_ACTIVATION_AND_REGULATION	Genes involved in eNOS activation and regulation	3	4.48E-02
	REACTOME_AMINO_ACID_AND_OLIGOPEPTIDE_SLC_TRANSPORTERS	Genes involved in Amino acid and oligopeptide SLC transporters	4	4.48E-02
	BIOCARTA_ATRBRCA_PATHWAY	Role of BRCA1, BRCA2 and ATR in Cancer Susceptibility	3	4.48E-02
GeneOntology Biological Processes	SIGNAL_TRANSDUCTION	Genes annotated by the GO term GO:0007165. The cascade of processes by which a signal interacts with a receptor, causing a change in the level or activity of a second messenger or other downstream target, and ultimately effecting a change in the functioni	32	3.90E-04
	CELLULAR_MACROMOLECULE_METABOLIC_PROCESS	Genes annotated by the GO term GO:0044260. The chemical reactions and pathways involving macromolecules, large molecules including proteins, nucleic acids and carbohydrates, as carried out by individual cells.	26	3.90E-04
	CELL_DEVELOPMENT	Genes annotated by the GO term GO:0048468. The process whose specific outcome is the progression of the cell over time, from its formation to the mature structure. Cell development does not include the steps involved in committing a cell to a specific fat	17	3.90E-04

(*Continued*)

GSEA Category	Gene Set Name	Description	#Genes in Overlap	FDR *q*-value
	PROTEIN_METABOLIC_PROCESS	Genes annotated by the GO term GO:0019538. The chemical reactions and pathways involving a specific protein, rather than of proteins in general. Includes protein modification.	27	3.90E-04
	APOPTOSIS_GO	Genes annotated by the GO term GO:0006915. A form of programmed cell death induced by external or internal signals that trigger the activity of proteolytic caspases, whose actions dismantle the cell and result in cell death. Apoptosis begins internally wi	14	5.68E-04
	PROGRAMMED_CELL_DEATH	Genes annotated by the GO term GO:0012501. Cell death resulting from activation of endogenous cellular processes.	14	5.68E-04
	RESPONSE_TO_STRESS	Genes annotated by the GO term GO:0006950. A change in state or activity of a cell or an organism (in terms of movement, secretion, enzyme production, gene expression, etc.) as a result of a stimulus indicating the organism is under stress. The stress is	14	2.68E-03
	TRANSPORT	Genes annotated by the GO term GO:0006810. The directed movement of substances (such as macromolecules, small molecules, ions) into, out of, within or between cells.	17	7.24E-03
	NEGATIVE_REGULATION_OF_CELLULAR_PROCESS	Genes annotated by the GO term GO:0048523. Any process that stops, prevents or reduces the frequency, rate or extent of cellular processes, those that are carried out at the cellular level, but are not necessarily restricted to a single cell. For example,	15	7.24E-03
	ACTIN_CYTOSKELETON_ORGANIZATION_AND_BIOGENESIS	Genes annotated by the GO term GO:0030036. A process that is carried out at the cellular level which results in the formation, arrangement of constituent parts, or disassembly of cytoskeletal structures comprising actin filaments and their associated prot	6	8.39E-03
	INTRACELLULAR_SIGNALING_CASCADE	Genes annotated by the GO term GO:0007242. A series of reactions within the cell that occur as a result of a single trigger reaction or compound.	14	2.01E-02
	RESPONSE_TO_EXTERNAL_STIMULUS	Genes annotated by the GO term GO:0009605. A change in state or activity of a cell or an organism (in terms of movement, secretion, enzyme production, gene expression, etc.) as a result of an external stimulus.	9	2.09E-02

(*Continued*)

GSEA Category	Gene Set Name	Description	#Genes in Overlap	FDR *q*-value
	VESICLE_MEDIATED_ TRANSPORT	Genes annotated by the GO term GO:0016192. The directed movement of substances, either within a vesicle or in the vesicle membrane, into, out of or within a cell.	7	2.15E-02
	INDUCTION_OF_ APOPTOSIS_BY_ INTRACELLULAR_SIGNALS	Genes annotated by the GO term GO:0008629. Any process induced by intracellular signals that directly activates any of the steps required for cell death by apoptosis.	3	2.35E-02
	POSITIVE_REGULATION_ OF_CELL_DIFFERENTIATION	Genes annotated by the GO term GO:0045597. Any process that activates or increases the frequency, rate or extent of cell differentiation.	3	2.46E-02
	ORGANELLE_ ORGANIZATION_AND_ BIOGENESIS	Genes annotated by the GO term GO:0006996. A process that is carried out at the cellular level which results in the formation, arrangement of constituent parts, or disassembly of any organelle within a cell.	11	2.51E-02
	REGULATION_OF_ APOPTOSIS	Genes annotated by the GO term GO:0042981. Any process that modulates the occurrence or rate of cell death by apoptosis.	9	2.92E-02
	REGULATION_OF_ PROGRAMMED_CELL_ DEATH	Genes annotated by the GO term GO:0043067. Any process that modulates the frequency, rate or extent of programmed cell death, cell death resulting from activation of endogenous cellular processes.	9	2.92E-02
	INDUCTION_OF_ APOPTOSIS_BY_ EXTRACELLULAR_SIGNALS	Genes annotated by the GO term GO:0008624. Any process induced by extracellular signals that directly activates any of the steps required for cell death by apoptosis.	3	1.86E-02
**Oncogenic signatures**	ALK_DN.V1_UP	Genes up-regulated in DAOY cells (medulloblastoma) upon knockdown of ALK [Gene ID = 238] gene by RNAi.	7	2.00E-02
	CYCLIN_D1_UP.V1_DN	Genes down-regulated in MCF-7 cells (breast cancer) over-expressing CCND1 [Gene ID = 595] gene.	7	2.83E-02
	RAF_UP.V1_DN	Genes down-regulated in MCF-7 cells (breast cancer) positive for ESR1 [Gene ID = 2099] MCF-7 cells (breast cancer) stably over-expressing constitutively active RAF1 [Gene ID = 5894] gene.	8	2.83E-02
	CTIP_DN.V1_DN	Genes down-regulated in MCF10A cells (breast cancer) upon knockdown of RBBP8 [Gene ID=RBBP8] gene by RNAi.	6	2.83E-02
	KRAS.LUNG_UP.V1_DN	Genes down-regulated in epithelial lung cancer cell lines over-expressing an oncogenic form of KRAS [Gene ID = 3845] gene.	6	2.83E-02

(*Continued*)

GSEA Category	Gene Set Name	Description	#Genes in Overlap	FDR *q*-value
	TBK1.DF_UP	Genes up-regulated in epithelial lung cancer cell lines upon over-expression of an oncogenic form of KRAS [Gene ID = 3845] gene and knockdown of TBK1 [Gene ID = 29110] gene by RNAi.	9	4.64E-02
	CSR_LATE_UP.V1_UP	Genes up-regulated in late serum response of CRL 2091 cells (foreskin fibroblasts).	7	4.64E-02
	ATF2_S_UP.V1_DN	Genes down-regulated in myometrial cells over-expressing a shortened splice form of ATF2 [Gene ID = 1386] gene.	6	4.64E-02
	ATF2_UP.V1_DN	Genes down-regulated in myometrial cells over-expressing ATF2 [Gene ID = 1386] gene.	6	4.64E-02
	IL15_UP.V1_DN	Genes down-regulated in Sez-4 cells (T lymphocyte) that were first starved of IL2 [Gene ID = 3558] and then stimulated with IL15 [Gene ID = 3600].	6	4.64E-02
	JNK_DN.V1_DN	Genes down-regulated in JNK inhibitor-treated (SP600125[PubChem = 8515]) keratinocytes.	6	4.64E-02
	NFE2L2.V2	Genes up-regulated in MEF cells (embryonic fibroblasts) with knockout of NFE2L2 [Gene ID = 4780] gene.	10	4.64E-02
	NRL_DN.V1_UP	Genes up-regulated in retina cells from NRL [Gene ID = 4901] knockout mice.	5	4.64E-02
	EGFR_UP.V1_DN	Genes down-regulated in MCF-7 cells (breast cancer) positive for ESR1 [Gene ID = 2099] and engineered to express ligand-activatable EGFR [Gene ID = 1956].	7	4.64E-02

Supplementary Table S4. miRNA differentially expressed in eEBV+ BL vs EBV+ PTLD (Mann-Whitney, p < 0.05; fold change > 2)

Target	*p*	FC (abs)	Regulation in BL	Accession
ebv-miR-BART1–3p	0.030654237	5.049484	up	nmiR00719.1
ebv-miR-BART3	0.025347317	7.0933237	up	nmiR00742.1
ebv-miR-BART4	0.017072659	3.614636	up	nmiR00743.1
ebv-miR-BART8	0.02085476	4.814401	up	nmiR00748.1
ebv-miR-BART9	0.030654237	7.128088	up	nmiR00749.1
ebv-miR-BART11-5p	0.036888424	4.2589674	up	nmiR00723.1
ebv-miR-BART17-3p	0.009087466	7.7453837	up	nmiR00729.1
ebv-miR-BART19-3p	0.003650433	6.251706	up	nmiR00733.1
ebv-miR-BART19-5p	0.030654237	4.281322	up	nmiR00734.1
ebv-miR-BART22	4.42E-02	10.655258	up	nmiR00741.1

Supplementary Table S5. EBV-encoded miRNA differentially expressed in eEBV+ BL vs EBV+ lymphadenitis (Mann-Whitney, $p < 0.05$; fold change > 2)

Target	*p*	FC (abs)	Regulation	Accession
ebv-miR-BART4	0.043819796	6.8236814	up	nmiR00743.1
ebv-miR-BART9	0.043819796	10.066428	up	nmiR00749.1
ebv-miR-BART11-5p	0.043819796	10.686326	up	nmiR00723.1

Supplementary Table S6. Targets of differentially expressed EBV-encoded miRNAs which are differentially expressed in EBV-positive vs. EBV-negative Burkitt lymphomas (extracted from VIRmiRNA database: http://crdd.osdd.net/servers/virmirna/)

TargetID	*p*	Regulation	FC
ABHD4	0.032205	down	–10.1553
ABTB2	0.023686	down	–5.19592
ARL5B	0.006763	down	–4.86802
ASF1A	0.049346	down	–4.20251
B4GALT5	0.013891	down	–10.5721
BAX	0.024799	down	–4.04725
BIVM	0.008456	down	–7.49489
BPTF	0.01317	down	–4.59085
BRCA1	0.043518	down	–3.03454
BSN	0.029684	down	–7.64984
CBFB	0.041061	down	–3.23621
CCDC117	0.041096	down	–3.76309
CDK8	0.004391	up	13.56905
CDKN1A	0.020664	down	–3.84729
CFL2	0.035167	down	–3.80119
CHIC2	0.043101	down	–8.1288
COMMD6	0.020806	down	–3.85141
CTDSPL2	0.027014	down	–4.73189
CUX1	0.023781	up	4.602538
CXXC4	0.003467	down	–9.26937
DAP	0.008188	up	8.716893
DDX3Y	0.026716	down	–5.07749
DDX46	0.021423	down	–6.07202
DENND5B	0.035247	down	–4.92635
DYRK2	0.01314	down	–3.77783
EXOC5	0.018976	down	–11.4029
FAM73A	0.04837	down	–3.77829
FANCF	4.11E-04	down	–17.1716
FCHO2	0.012829	down	–8.74379
FMR1	0.006853	down	–6.25687
FNDC3A	0.023775	down	–5.22722
FTO	0.030624	down	–7.02913
FZD3	0.002384	down	–8.65198
GEMIN4	0.049144	up	5.885448
GRIA2	0.023012	down	–5.22994

(*Continued*)

TargetID	*p*	Regulation	FC
HIP1	0.009177	down	–8.16208
HIPK1	0.011137	down	–4.67427
HMGCL	0.045303	up	6.049655
HSBP1	0.023963	up	5.067395
HSDL1	0.024297	up	10.83806
IMPA1	0.040138	down	–3.15563
JAG1	0.025339	up	7.14392
KIAA0232	0.021247	down	–5.30728
KIAA1432	0.002462	down	–9.54355
KLHL18	0.034864	down	–5.14205
LIN28B	6.35E-04	down	–49.5019
LZIC	0.015731	up	11.10423
MARK3	0.03183	up	3.733798
MDM4	0.020036	down	−4.97414
MGAT5	0.026215	down	–8.16219
MITF	0.008565	down	–5.80468
MOBKL1A	0.049224	down	–2.60761
MSC	0.02498	down	–7.20591
MTMR1	0.043632	down	–4.28474
NAPEPLD	0.016193	down	–5.52392
NCK1	0.026144	down	–5.21929
NDUFA7	0.028335	up	10.4013
NOS3	0.032316	up	10.27049
PAICS	0.01897	down	–4.02996
PAPOLG	0.049258	up	4.451463
PLAG1	0.016248	down	–4.61203
PPARD	0.041682	down	–3.33435
RAB12	0.048274	up	4.350109
RAG1	0.006303	down	–8.12452
RBAK	0.012248	down	–11.6937
RBM38	0.025088	down	–3.45653
RC3H1	0.040329	down	–3.75174
RCOR3	0.024651	down	–3.88355
SACS	0.036533	down	–4.22353
SAMD9	0.049037	down	–3.80753
SBF2	0.031912	down	–4.51189
SC5DL	0.03289	down	–3.69287

(*Continued*)

TargetID	*p*	Regulation	FC
SEL1L	0.009018	down	–15.2552
SEMA3D	0.033018	down	–6.09483
SLAMF1	0.040316	down	–7.12938
SLC16A14	0.032562	down	–3.67477
SLC30A6	0.033728	down	–3.34784
SLC37A3	0.045926	down	–3.25096
SMARCC1	0.024836	up	7.415126
SMEK2	0.043172	down	–2.45191
TBCC	0.008943	down	–6.44556
TET2	0.039258	down	–4.90178
TMCC1	0.041383	down	–3.56969
TMEM20	0.014023	down	–6.63398
TMEM39A	0.036486	down	–4.6623
TNKS2	0.043024	down	–3.76089
TRAM1	0.02541	down	–8.24787
TRIB1	0.045444	up	4.113507
TRIM23	0.041023	down	–3.35212
TTC27	0.039125	up	6.430929
UAP1L1	0.035453	down	–3.52568
USPL1	0.010011	down	–6.44799
WASL	0.01314	down	–6.63221
ZFP3	0.039059	up	6.339253
ZFYVE26	0.014627	up	4.246381
ZNF12	0.039514	down	–4.237
ZNF24	0.043159	down	–6.47336
ZNF264	0.043337	down	–5.4025
ZNF268	0.004841	down	–6.49412
ZNF28	0.048287	down	–4.54129
ZNF493	0.039465	down	–3.10945
ZNF673	0.03622	down	–3.43494
ZNF714	0.038507	down	–4.04006

Supplementary Table S7. GSEA analysis carried on the targets of differentially expressed EBV-encoded miRNAs which are differentially expressed in EBV+ vs. EBV- BL cases revealed significant enrichment in several programs and pathways

Gene Set Name	# Genes in Gene Set (K)	Description	# Genes in Overlap (k)	k/K	*p*-value	FDR *q*-value
MTOR	193	Genes up-regulated in CEM-C1 cells (T-CLL) by everolimus [PubChem = 6442177], an mTOR pathway inhibitor.	7	0.0363	2.91E-07	3.30E-05
STK33	293	Genes up-regulated in NOMO-1 and SKM-1 cells (AML) after knockdown of STK33 [Gene ID = 65975] by RNAi.	8	0.0273	3.49E-07	3.30E-05
LTE2	196	Genes down-regulated in MCF-7 cells (breast cancer) positive for ESR1 [Gene ID = 2099] MCF-7 cells (breast cancer) and long-term adapted for estrogen-independent growth.	5	0.0255	8.36E-05	3.16E-03
TBK1	287	Genes down-regulated in epithelial lung cancer cell lines upon over-expression of an oncogenic form of KRAS [Gene ID = 3845] gene and knockdown of TBK1 [Gene ID = 29110] gene by RNAi.	5	0.0174	4.88E-04	1.54E-02
CYCLIN D1	190	Genes up-regulated in MCF-7 cells (breast cancer) over-expressing a mutant K112E form of CCND1 [Gene ID = 595] gene.	4	0.0211	9.10E-04	1.86E-02
P53	192	Genes down-regulated in NCI-60 panel of cell lines with mutated TP53 [Gene ID = 7157].	4	0.0208	9.46E-04	1.86E-02
TGFB	192	Genes up-regulated in a panel of epithelial cell lines by TGFB1 [Gene ID = 7040].	4	0.0208	9.46E-04	1.86E-02
HOXA9	194	Genes up-regulated in MOLM-14 cells (AML) with knockdown of HOXA9 [Gene ID = 3205] gene by RNAi vs controls.	4	0.0206	9.83E-04	1.86E-02
NUCLEIC ACID METABOLISM	1244	Genes annotated by the GO term GO:0006139. The chemical reactions and pathways involving nucleobases, nucleosides, nucleotides and nucleic acids.	16	0.0129	1.86E-08	1.52E-05
RNA METABOLIC PROCESS	841	Genes annotated by the GO term GO:0016070. The chemical reactions and pathways involving RNA, ribonucleic acid, one of the two main type of nucleic acid, consisting of a long, unbranched macromolecule formed from ribonucleotides joined in 3′, 5′-phosphodiester linkage.	11	0.0131	3.13E-06	1.61E-04

(*Continued*)

Gene Set Name	# Genes in Gene Set (K)	Description	# Genes in Overlap (k)	k/K	*p*-value	FDR *q*-value
REGULATION OF GENE EXPRESSION	673	Genes annotated by the GO term GO:0010468. Any process that modulates the frequency, rate or extent of gene expression. Gene expression is the process in which a gene's coding sequence is converted into a mature gene product or products (proteins or RNA). This includes the production of an RNA transcript as well as any processing to produce a mature RNA product or an mRNA (for protein-coding genes) and the translation of that mRNA into protein. Some protein processing events may be included when they are required to form an active form of a product from an inactive precursor form.	10	0.0149	2.95E-06	1.61E-04
REGULATION OF METABOLIC PROCESS	799	Genes annotated by the GO term GO:0019222. Any process that modulates the frequency, rate or extent of the chemical reactions and pathways within a cell or an organism.	12	0.015	2.53E-07	2.61E-05
PROGRAMMED CELL DEATH	432	Genes annotated by the GO term GO:0012501. Cell death resulting from activation of endogenous cellular processes.	8	0.0185	6.23E-06	2.52E-04
APOPTOSIS	431	Genes annotated by the GO term GO:0006915. A form of programmed cell death induced by external or internal signals that trigger the activity of proteolytic caspases, whose actions dismantle the cell and result in cell death. Apoptosis begins internally with condensation and subsequent fragmentation of the cell nucleus (blebbing) while the plasma membrane remains intact. Other characteristics of apoptosis include DNA fragmentation and the exposure of phosphatidyl serine on the cell surface.	8	0.0186	6.13E-06	2.52E-04
NEGATIVE REGULATION OF TRANSCRIPTION	188	Genes annotated by the GO term GO:0016481. Any process that stops, prevents or reduces the frequency, rate or extent of transcription.	7	0.0372	2.44E-07	2.61E-05
INDUCTION OF APOPTOSIS BY INTRACELLULAR SIGNALS	24	Genes annotated by the GO term GO:0008629. Any process induced by intracellular signals that directly activates any of the steps required for cell death by apoptosis.	4	0.1667	2.44E-07	2.61E-05

Supplementary Table S8. Genes differentially expressed upon transfection of Akata cell line with BART6–3p inhibitor

probe_ID	Gene_Symbol	Regulation in BART6–3p Inhibitor	*p*-Value	Fold Change (log2)
16978164	ADH5	Down	0.0028223	–2.280493677
16802791	ADPGK-AS1	Up	0.005855549	2.203000605
16723944	ALKBH3	Down	0.003454235	–2.051026017
17008404	APOBEC2	Down	0.007839291	–2.086709917
16695535	ARHGAP30	Down	0.008130218	–1.604154684
16799567	BAHD1	Down	0.00814453	–1.700475439
16758879	BRI3BP	Down	0.004512641	–1.936326563
16794397	C14orf57	Up	0.009793379	2.407289505
16712148	C1QL3	Up	0.003611382	1.808043987
17110849	CACNA1F	Down	0.006116351	–1.73877129
16969686	CCDC109B	Down	0.00752574	–1.617655502
17051255	CCDC136	Down	0.001785038	–1.78460066
16991839	CCNG1	Down	0.008981167	–2.08486408
17126214	CDKN2B-AS1	Down	0.008828001	–2.082891703
16841657	CDRT1	Up	0.008488477	2.274805009
16981889	CLDN22	Up	2.46821E-4	2.423785597
16921664	CXADR	Down	0.005969327	–1.739408899
17037538	DAXX	Down	0.004504517	–1.603255082
17060852	DGAT2L7	Up	0.009169811	1.588970646
16765845	DGKA	Down	0.003315417	–1.93541041
16820927	DHODH	Down	0.001433172	–1.609117255
16912597	DNMT3B	Down	0.004978167	–2.299726605
16679569	EFCAB2	Down	0.007230868	–2.53817147
16764602	FAM186A	Up	0.001795151	2.359458327
16879205	FAM82A1	Up	0.001431876	2.134093851
16917061	FERMT1	Up	0.005988875	1.711756963
17016995	FLOT1	Down	0.004962849	–1.919253051
17031666	FLOT1	Down	0.004962849	–1.919253051
17039163	FLOT1	Down	0.004962849	–1.919253051
17041731	FLOT1	Down	0.004962849	–1.919253051
17024374	FUCA2	Up	0.008120241	2.363443762
16991261	G3BP1	Down	0.0093378	–2.176404625
16859795	GDF15	Down	0.004859705	–2.047232419
17095111	GNAQ	Up	0.004106787	2.0530698
16991192	GPX3	Down	0.001247659	–1.961835593
17116962	GYG2P1	Down	0.004306936	–1.952549607

(*Continued*)

probe_ID	Gene_Symbol	Regulation in BART6–3p Inhibitor	*p*-Value	Fold Change (log2)
16884629	IL1RN	Down	0.004725387	–1.710031003
16727045	KAT5	Down	0.007698467	–1.926847488
16697492	KCNT2	Up	0.00839957	2.030667022
16755008	LINC00615	Up	0.00430693	2.038129702
16742115	LIPT2	Down	0.007978634	–2.065563858
16666881	LMO4	Down	0.006964794	–2.336911738
16965004	LOC100288520	Up	0.009280462	1.615047142
16798134	LOC100506948	Down	0.002453247	–1.959390327
16758564	LOC100507091	Up	0.005690878	1.897721112
17117732	LOC124685	Up	0.005187913	2.102300555
16883826	LOC150568	Up	0.009010807	1.680562094
17045130	LOC401324	Up	0.008512338	2.225022018
16803008	LOC440288	Down	1.3434535E-4	–1.759779237
16923745	LOC642852	Up	0.001947448	1.686120749
16962264	MAGEF1	Down	0.007046318	–1.660783187
16930264	MGAT3	Down	0.008222118	–1.648295939
16783662	MIA2	Up	0.002369667	1.924192399
16979101	MIR302B	Up	0.006693444	2.132579535
17092879	MIR31	Up	0.009312762	1.951798201
16738774	MRPL16	Down	0.009286948	–2.449375868
16837754	MRPS7	Down	0.001457722	–2.114717186
16921724	NCAM2	Up	0.004233315	1.694271773
16807613	NDUFAF1	Down	0.001849802	–1.998230934
16691262	NRAS	Down	0.007354182	–2.517931044
16894782	NT5C1B-RDH14	Down	0.002322084	–2.46295619
16679785	OR2M1P	Up	0.009169838	1.977240622
16781456	OR4K15	Up	0.007179865	1.964963764
16781431	OR4N2	Up	0.001755431	2.039425552
16939142	OXSR1	Down	0.008119614	–2.308969349
16740914	PC	Down	1.3885966E-4	–2.020653248
16757710	PEBP1	Down	8.669243E-4	–2.060452029
16996433	PLK2	Up	0.009802544	2.029658824
16836476	PPM1E	Down	0.004244627	–1.642680049
16913065	PROCR	Up	0.002898674	2.060634404
16675578	PTPRC	Down	0.005187966	–1.900309935
16759592	PXMP2	Down	0.002662436	–2.03574498
17118432	RBSG2	Down	0.00669313	–1.949072301

(*Continued*)

probe_ID	Gene_Symbol	Regulation in BART6–3p Inhibitor	*p*-Value	Fold Change (log2)
16837101	RGS9	Down	0.001643967	–2.491684616
17123724	RNF185	Down	0.001432825	–1.807868049
16775377	RNY1P8	Up	0.00818199	1.70498614
16877019	RRM2	Down	0.004746406	–1.625113256
16972425	SAP30	Down	0.004736937	–2.213891
16691023	SLC16A1	Down	0.008399573	–1.812251657
17061634	SLC26A3	Up	0.005695865	1.94827494
16721375	SMPD1	Down	0.004083642	–1.881971419
16773485	SNORD102	Down	0.002755861	–2.010462105
16718263	SORCS1	Up	0.004802579	2.204250902
17101799	SYAP1	Down	0.006519921	–2.070559859
16906509	TMEM194B	Down	0.001957257	–1.66842439
17051524	TMEM209	Up	0.008772072	1.974466562
16735639	TMEM41B	Down	0.007525738	–2.132966787
16827248	TRADD	Down	0.001676114	–1.931642503
17084359	TRBV20OR9–2	Up	0.006652342	2.140173018
16724683	TRIM49B	Up	0.002566569	1.726456299
16978278	TRMT10A	Down	0.002647853	–2.382619321
17091877	TUBBP5	Up	0.00723084	1.607002392
17093325	UBAP2	Down	0.007266358	–2.189597756
17056896	VPS41	Down	0.002822304	–1.667562448
17011755	WISP3	Up	0.002541296	1.624920264
16918137	ZNF337	Down	0.00416075	–1.862966627
